# The Composite Digital Therapeutic Index (cDTI): A Multidimensional Framework and Proof-of-Concept Application to FDA-Authorized Treatments

**DOI:** 10.7759/cureus.83886

**Published:** 2025-05-11

**Authors:** Shaheen E Lakhan

**Affiliations:** 1 Medical Office, Click Therapeutics, Inc., New York, USA; 2 Department of Neurology, Western University of Health Sciences, Pomona, USA; 3 Department of Neurology, A.T. Still University School of Osteopathic Medicine in Arizona, Mesa, USA; 4 Department of Neurology, Morehouse School of Medicine, Atlanta, USA; 5 Department of Bioscience, Global Neuroscience Initiative Foundation, Miami, USA

**Keywords:** composite score, digital therapeutics, efficacy, engagement, health technology assessment, prescription digital therapeutics, safety

## Abstract

Introduction

Prescription digital therapeutics (PDTs) are an emerging class of regulated software-based interventions with increasing FDA authorizations. However, there is no standardized framework to evaluate and benchmark their overall therapeutic value.

We aimed to develop and validate a Composite Digital Therapeutic Index (cDTI) framework. This proof-of-concept application integrates efficacy, engagement, quality of evidence, and safety profile from registrational trials to compare PDTs using publicly available regulatory summaries, registries, and study readouts.

Methods

We developed a scoring framework incorporating four domains (efficacy, engagement, evidence quality, and safety), each quantified and combined into a composite index. Efficacy was calculated using standardized mean difference (SMD; Hedges' g) adjusted for statistical significance. Engagement was based on the mean proportion of patients achieving therapeutic module completion. Evidence quality was graded using an adapted American Academy of Neurology Class of Evidence framework. Safety was assessed based on treatment-emergent adverse events (TEAEs) using a hierarchical penalty system. In a proof-of-concept application of the framework, we applied cDTI to two FDA-cleared first- and second-generation PDTs, reSET-O for opioid use disorder (Pear Therapeutics, Boston, MA, USA) and CT-132 for episodic migraine (Click Therapeutics, New York, NY, USA), respectively.

Results

CT-132 achieved a cDTI score of 0.296, substantially (1287%) higher than reSET-O, which scored 0.023. CT-132’s superior performance was driven by a statistically significant efficacy outcome (adjusted SMD 0.33), high engagement (89.7%), Class I evidence quality with sham and double-blind, and no treatment-related adverse events. In contrast, reSET-O showed a non-significant primary outcome (adjusted SMD 0.0895), moderate engagement (63.0%), Class III evidence quality without patient blind and usual-care control, and missing systematic safety reporting requiring a conservative penalty.

Discussion

The cDTI successfully differentiated the overall therapeutic profiles of two PDTs across multiple clinical and usability domains. This multidimensional approach highlights how digital therapeutics with similar regulatory status can differ meaningfully in trial quality, efficacy, engagement, and safety. The cDTI offers a transparent, reproducible method for comparing PDTs and may aid stakeholders including providers, payers, regulators, and patients in decision-making about digital therapeutic adoption and coverage.

Conclusion

The cDTI provides a reproducible framework to evaluate PDTs based on clinical, usability, and safety parameters. Future work aims to expand the framework across all FDA-cleared PDTs and explore incorporation of real-world effectiveness, equity, and cost-effectiveness domains.

## Introduction

Prescription digital therapeutics (PDTs) are regulated, software-based interventions delivered via smartphones to provide evidence-based treatments for a variety of medical conditions under clinician supervision [1]. Unlike wellness apps or consumer-facing health platforms, PDTs undergo regulatory review and clearance by the U.S. Food and Drug Administration (FDA). As of April 2025, more than a dozen PDTs have been authorized through the 510(k) and De Novo pathways, targeting conditions such as opioid use disorder [2], insomnia [3], major depressive disorder [4], and migraine [5].

Despite this regulatory momentum, the field lacks a standardized, quantitative framework for evaluating and comparing PDTs across clinical indications. Health systems, payers, and clinicians face challenges due to the absence of benchmark tools that assess overall therapeutic value beyond single-trial efficacy. Existing health technology assessments (HTAs) frequently rely on qualitative grading systems or domain-specific evaluations [6], but no composite scoring system exists to synthesize these domains into a unified index tailored to digital therapeutics.

To address this gap, we developed the Composite Digital Therapeutic Index (cDTI), a next-generation, multidomain scoring framework that integrates clinical efficacy, patient engagement, evidence quality, and safety profile into a unified composite score. The cDTI is designed to capture the multidimensional therapeutic value of PDTs and to support evidence-based comparisons across digital interventions of varying designs and technological maturity.

In this proof-of-concept study, we both introduce and validate the cDTI framework by applying it to two FDA-cleared PDTs representative of different generations of digital therapeutic development [1]: reSET-O (Pear Therapeutics, Boston, MA, USA) [1], an early first-generation PDT for opioid use disorder, and CT-132 (Click Therapeutics, New York, NY, USA) [4], a second-generation PDT for episodic migraine. This approach illustrates the cDTI's ability to differentiate therapeutic value across evolving classes of PDTs and to benchmark innovation in the digital health field.

## Materials and methods

Framework development and rationale

The cDTI was developed to provide a multidimensional, reproducible assessment of the therapeutic value of PDTs. Four key domains were selected - efficacy, engagement, evidence quality, and safety - based on their relevance to regulatory approval, clinical decision-making, and real-world adoption. The cDTI is intended to synthesize these domains into a single, interpretable metric, enabling structured comparisons across PDTs of differing technological maturity.

Efficacy captures the clinical magnitude of benefit, engagement reflects real-world usability and adherence, evidence quality ensures methodological rigor, and safety accounts for potential treatment-related harm. Together, these domains align with HTA standards used by organizations such as Grading of Recommendations Assessment, Development and Evaluation (GRADE) [7], the Institute for Clinical and Economic Review (ICER) [8], and international regulatory bodies [9].

The primary aim of this study was to generate and operationalize the cDTI framework. As a proof-of-concept validation, we applied the cDTI to two FDA-cleared PDTs representing different generational eras: reSET-O (PDT 1.0) and CT-132 (PDT 2.0). This allowed evaluation of the cDTI's ability to distinguish therapeutic value across historical and contemporary digital therapeutics.

Data sources

Data were systematically extracted from publicly available regulatory documents (FDA De Novo, 510(k), and clinician brief summaries) [1,5,10], prospective clinical trial registries (e.g. ClinicalTrials.gov) [11,12], and peer-reviewed pivotal trial publications [13-15]. When available, supplementary materials such as therapeutic and health technology assessments (e.g., ICER) [16,17] and FDA special designations [18] were referenced to validate outcomes.

Scoring domains

Efficacy

Efficacy was quantified by calculating the standardized mean difference (SMD) between the treatment and control arms on the trial’s prespecified primary clinical endpoint. Hedges’ g, a small-sample bias-corrected version of Cohen’s d, was used to ensure greater statistical accuracy, particularly for trials with modest sample sizes [19]. For trials reporting multiple outcomes, the prespecified primary endpoint was prioritized based on trial registration, regulatory filings, or explicit designation in publications. If no primary endpoint was specified, the first major clinical outcome presented was selected according to a prespecified hierarchy. To standardize interpretation across outcome types, directionality was normalized so that positive SMD values always indicated greater therapeutic benefit.

A tiered adjustment based on p-value was applied to the SMD: very highly statistically significant at p<0.001 corresponded to a 1.15× multiplier, highly statistically significant at 0.001≤p<0.01 corresponded to a 1.10× multiplier, statistically significant at 0.01≤p<0.05 resulted in no adjustment (1.00×), and not statistically significant at p≥0.05 was conservatively penalized with a 0.50× multiplier. This adjustment rewarded statistical robustness while conservatively penalizing uncertainty. Although heuristic, the p-value multipliers were prospectively prespecified, bounded by a maximum adjustment cap of +15%, and designed to avoid disproportionate inflation of efficacy contributions. When exact p-values were unavailable, significance was inferred from confidence intervals or reported interpretations; otherwise, a non-significant assumption (p≥0.05) was applied.

While traditional meta-analytic methods treat effect size and statistical significance separately, therapeutic and health technology assessment frameworks such as ICER and GRADE systematically incorporate both magnitude of effect and precision (p-values, confidence intervals) into strength of evidence evaluations [7,8]. Our use of tiered p-value adjustments in the cDTI similarly aims to reward statistically robust findings while conservatively downweighting uncertain results, in alignment with these established practices. The adjustment is intended to optimize the relative weighting of efficacy evidence across heterogeneous digital interventions, rather than to replace traditional hypothesis testing or meta-analytic effect size synthesis.

No minimal clinically important difference (MCID) thresholds were used, reflecting the cDTI’s purpose as a cross-condition benchmarking tool rather than a condition-specific utility score. All efficacy scoring was performed prospectively using these procedures, without post hoc adjustment based on therapeutic area, sponsorship, or perceived clinical importance, ensuring objectivity and reproducibility across all evaluated clinical products. The efficacy rules are summarized in Table 1.

**Table 1:** Summary of Efficacy Scoring Rules for cDTI Calculation This author-created table outlines the key procedural rules applied to efficacy scoring within the Composite Digital Therapeutic Index (cDTI) framework, including endpoint prioritization, directionality normalization, p-value-based adjustments to standardized mean differences (SMDs), and handling of missing or nonsignificant results. Minimal Clinically Important Difference (MCID) was not used in this framework, reflecting the cross-condition benchmarking design of the cDTI. These rules ensure consistency, objectivity, and reproducibility across evaluated digital therapeutics.

Rule	Description
Primary Endpoint Prioritization	Prespecified endpoint prioritized; if absent, first major clinical outcome selected per prespecified hierarchy.
Directionality Normalization	Positive SMD values standardized to indicate greater therapeutic benefit.
p-Value Adjustment for Very Highly Statistically Significant (p<0.001)	SMD multiplied by 1.15.
p-Value Adjustment for Highly Statistically Significant (0.001≤p<0.01)	SMD multiplied by 1.10.
p-Value Adjustment for Statistically Significant (0.01≤p<0.05)	SMD multiplied by 1.00 (no adjustment).
p-Value Adjustment for Not Statistically Significant (p≥0.05)	SMD multiplied by 0.50 (penalized).
Handling Missing p-Values	Significance inferred from confidence intervals or interpretation; if unavailable, assumed non-significant.
MCID Thresholds	No MCID thresholds applied; cDTI intended as cross-condition benchmarking tool.
Objectivity Assurance	Scoring performed prospectively without post hoc adjustment based on therapeutic area or sponsor.

Engagement

Engagement was defined as the proportion of participants who completed the digital intervention or achieved adherence to core therapeutic modules, tasks, or sessions, consistent with definitions used in behavioral and digital intervention research [20-22].

Engagement data were systematically extracted from trial publications and regulatory summaries following a hierarchical preference order designed to prioritize objective, reproducible measures. First, objective module completion data, such as the number or percentage of therapeutic modules completed, were used when available. If objective completion metrics were not reported, digital usage data (e.g., app logins, session counts, or time-on-task) were used as a proxy for engagement. If neither objective module nor digital usage metrics were available, clinician-assessed adherence rates, based on investigator evaluations or patient self-report during study visits, were considered. Finally, if none of the above were reported, study-defined “treatment completion” rates, reflecting participants who completed the full duration of the study irrespective of digital engagement, were used as a last-resort proxy measure.

Where engagement was not explicitly reported, a conservative imputation value of 0.50 was assigned. This assumption reflects a neutral midpoint to minimize bias introduced by missing data.

Evidence Quality

Evidence quality was assessed using an adapted application of the American Academy of Neurology Class of Evidence (AAN CoE) framework [23], modified to accommodate the unique characteristics and heightened methodological standards appropriate for PDT trials. Trials were classified based on methodological rigor (Table 2). Several adaptations were made to align the AAN CoE framework with the specific challenges of PDT research (Table 3). To ensure consistency, frequent PDT trial designs were classified under the adapted CoE framework (Table 4).

**Table 2:** Evidence Quality Classification Adapted from American Academy of Neurology Class of Evidence and Assigned Scores This author-created table summarizes the classification system used to evaluate the evidence quality score for the Composite Digital Therapeutic Index (cDTI). Each class reflects increasing levels of potential bias, with Class I representing the highest rigor (e.g., randomized, double-blind controlled trials) and Class IV indicating the lowest evidentiary value (e.g., non-controlled or observational studies). The numerical scores are used in the cDTI calculation to quantify evidence quality.

Class of Evidence	Score	Definition
Class I	1.00	Randomized, controlled trials conducted in a representative population with substantially equivalent baseline characteristics (or appropriate statistical adjustment), employing concealed allocation, clearly defined primary outcomes, explicit inclusion and exclusion criteria, ≥80% retention of enrolled patients, and true double-blinding, defined as masking of both the patient and the outcome assessor to treatment allocation. Sham or digital placebo-controlled designs were accepted if constructed to minimize expectancy and engagement biases while withholding active therapeutic content.
Class II	0.75	Randomized controlled trials conducted in a representative population with concealed allocation, defined primary outcomes, explicit eligibility criteria, and ≥80% retention, but lacking full double-blinding. Trials were classified as Class II if they maintained masked outcome assessment or used objective primary outcomes unlikely to be influenced by patient knowledge of treatment assignment.
Class III	0.50	Controlled trials lacking adequate randomization, masking, or objective outcome assessment, including natural history controls, patient-as-own-control designs, and waitlist control trials.
Class IV	0.25	Studies not meeting the criteria for Class I-III, including single-arm pre-post studies, expert opinion, or consensus reports.

**Table 3:** Adaptations to the American Academy of Neurology Class of Evidence Framework for Evidence Quality Score This author-created table outlines specific modifications made to the standard American Academy of Neurology Class of Evidence framework to account for the unique methodological considerations in digital therapeutic trials, including blinding, control design, engagement metrics, and adaptive features. These adaptations ensure appropriate grading of trial rigor for software-based interventions.

Adaptation	Description
Requirement for True Double-Blinding	To qualify for Class I evidence, trials were required to implement true double-blinding, meaning both the patient and the outcome assessor were unaware of treatment assignment. This minimizes expectancy and engagement biases inherent in interactive digital interventions.
Acceptance of Sham/Digital Controls	Sham or digital controls were accepted if constructed to control for expectancy and engagement without delivering therapeutic content.
Retention Versus Engagement Clarification	The ≥80% threshold applied specifically to trial completion and outcome assessment, distinct from in-app engagement metrics, which were evaluated separately.
Adaptive/Iterative Digital Designs	Trials with adaptive digital interventions were evaluated based on the methodological rigor of the prespecified pivotal phase, regardless of subsequent content updates.

**Table 4:** Classification of Common Digital Therapeutic Trial Designs Under the Adapted Evidence Quality Framework This author-created table presents frequent trial designs used in digital therapeutic development and their corresponding evidence classification under the adapted American Academy of Neurology Class of Evidence framework. Class assignments were based on trial design features such as control type, blinding, and methodological rigor.

Trial Design	Adapted Class of Evidence	Rationale
Waitlist-Controlled Trials	Class III	Lacks active comparator and carries a high risk of expectancy bias.
Usual Care-Controlled Trials	Class II or III	Assigned Class II if randomization and objective or masked outcome assessment were rigorous; downgraded to Class III if methodological deficiencies were present.
Single-Arm Pre-Post Studies	Class IV	No concurrent control group; limited internal validity.
Active Comparator Trials	Class I or II	Classified as Class I if double-blinded; Class II if single-blinded or lacking participant blinding.
Decentralized or Virtual Trials	Variable (Class I–IV)	Graded based on methodological quality (e.g., randomization, blinding, objective measurement) regardless of delivery modality.

Additional grading rules were applied to ensure consistency and methodological rigor. When discrepancies existed between peer-reviewed publications and regulatory summaries, the more conservative (lower) classification was assigned. In hybrid study designs, such as randomized controlled phases followed by open-label extensions, the class was determined solely based on the pivotal randomized controlled trial phase. For non-inferiority or equivalence trials, strict adherence to comparator similarity, eligibility matching, and observed-case analyses was required; failure to meet these criteria resulted in automatic downgrading by one class.

Numerical scores (1.0, 0.75, 0.5, 0.25) were assigned to facilitate interpretability, proportional weighting, and alignment with other evaluation frameworks such as GRADE [7]. The scale reflects meaningful differences in trial quality, although the specific values remain heuristic.

Safety

Safety was assessed based on treatment-emergent adverse event (TEAE) data reported in pivotal clinical trials and regulatory summaries, using a hierarchical extraction and penalty system to quantify potential harm attributable to the PDT (summarized in Table 5).

**Table 5:** Safety Data Extraction Hierarchy and Penalty Application in cDTI Calculation This author-created table outlines the hierarchical approach for extracting safety data and applying penalties in the Composite Digital Therapeutic Index (cDTI) framework. Safety penalties were subtracted from the denominator of the cDTI formula, such that higher penalties correspond to greater safety concerns and lower overall therapeutic scores. PDT: prescription digital therapeutics; TEAE: treatment-emergent adverse events

Safety Data Type	Extraction Rule	Penalty Application
PDT-related TEAE rate	Events adjudicated or attributed directly to the PDT	Raw incidence rate applied as penalty
ΔTEAE (difference in treatment vs. control TEAEs)	Used if PDT-related TEAE rate unavailable	Difference applied directly as penalty
Treatment-only TEAE rate	Used if no control arm or control safety data available	Treatment arm TEAE rate applied as penalty
No TEAE data reported	Default imputed penalty of 0.20	Direct assignment
Negative ΔTEAE or PDT-related TEAE rate	If fewer events in treatment than control	Safety penalty set to zero (no bonus)
Serious Adverse Events (SAEs) plausibly related to PDT	Hospitalization, life-threatening events, etc.	Additional penalty of 0.50 applied

Data extraction followed a prespecified hierarchy that was applied consistently across all PDTs. First, PDT-related TEAE rates were used, defined as events adjudicated or attributed directly to the PDT by investigators or regulators. If these were unavailable, the difference between overall TEAE rates in the treatment and control arms (ΔTEAE) was extracted. If ΔTEAE data were also unavailable, the overall TEAE rate in the treatment arm alone was used. If no TEAE data were reported, a conservative default safety penalty of 0.20 was imputed.

Penalty calculation rules were similarly standardized. For PDT-related TEAE rates, the raw incidence rate was applied directly as the penalty. For ΔTEAE, the difference between treatment and control arm rates was used. For treatment-only TEAEs, the raw treatment TEAE rate was applied as the penalty. If the calculated ΔTEAE or PDT-related TEAE rate was negative (i.e., the treatment arm exhibited fewer adverse events than the control arm), the safety penalty was set to zero. No bonus was awarded for apparent safety benefit, maintaining a conservative, risk-averse stance.

In trials reporting serious adverse events (SAEs) plausibly attributable to the PDT (e.g., hospitalization, life-threatening conditions), an automatic additional penalty of 0.50 was imposed, independent of TEAE rate differences. This safety scoring framework reflects trial-based tolerability under controlled conditions. Raw incidence rates were applied directly without normalization to maintain fidelity to reported data. If discrepancies arose between trial publications and regulatory summaries, the more conservative (higher) safety rate was applied.

Composite index calculation and radar plots

The final cDTI score was calculated using the formula:

$\text{cDTI} = \text{Efficacy} \times \text{Engagement} \times \text{Evidence Quality} \times (1 - \text{Safety Penalty})$

The cDTI is scaled from 0 to 1, where higher values represent greater overall therapeutic value. A score of 0 indicates no measurable efficacy, poor engagement, low-quality evidence, and/or significant safety concerns, whereas a score of 1 would indicate an ideal profile across all domains.

Efficacy (SMD using Hedges' g with statistical significance adjustment) and cDTI composite scores were manually calculated and cross-validated using Microsoft Excel (Microsoft Corp., Redmond, WA, USA). All calculations used basic algebraic operations and effect size formulas without the need for complex statistical modeling.

To support comparative benchmarking, radar plots were generated in Microsoft Excel to display individual scores for efficacy, engagement, evidence quality, and safety. This visual “therapeutic fingerprint” highlights the strengths and limitations of each PDT in a way that is intuitive for both technical and non-technical audiences

## Results

The cDTI was applied to two FDA-cleared PDTs, reSET-O and CT-132, to illustrate the scoring framework and benchmark therapeutic value across generations of digital therapeutics. Individual domain scores and final composite indices are summarized below.

reSET-O

Efficacy

The pivotal trial for reSET-O reported a mean difference of approximately 5.5 days in the primary endpoint of longest continuous abstinence compared to treatment as usual, with a total sample size of 170 patients, corresponding to a SMD (Hedges’ g) of 0.179 [1,10,12,14,15]. The primary endpoint was not statistically significant (p=0.214), qualifying for the p≥0.05 adjustment tier. After applying the penalty multiplier of 0.50, the adjusted efficacy score was 0.0895.

Engagement

There was no protocol-specified adherence threshold in the pivotal trial. Engagement was moderate, with patients completing a mean of 42.2 core modules out of 67 available across the 12-week intervention [14,15], corresponding to a core module completion rate of approximately 63%. Following the cDTI framework, the engagement rate was normalized to a 0-1 scale, yielding an engagement score of 0.630.

Evidence Quality

The pivotal trial was a randomized, usual-care controlled study without patient blinding (open-label) [12,14,15]. Outcome assessment relied on objective biological measures (urine drug screens) for the primary endpoint. Randomization procedures, defined eligibility criteria, and ≥80% patient retention were achieved. Under the adapted cDTI framework, the study was classified as Class III evidence, corresponding to an evidence quality score of 0.50.

Safety

Device-attributable safety outcomes were not systematically reported in the pivotal trial [12,14,15]. No TEAEs or ΔTEAEs were provided. Following predefined cDTI imputation rules for missing safety data, a default penalty of 0.20 was applied.

cDTI

The composite index for reSET-O was calculated as: 

$\text{cDTI} = 0.0895 \times 0.630 \times 0.50 \times (1 - 0.20) = 0.023$. 

Thus, reSET-O achieved a cDTI score of 0.023.

CT-132

Efficacy

The ReMMi-D pivotal trial of CT-132 reported a mean improvement of approximately 0.9 monthly migraine days over sham digital control with total sample size in the intention to treat (ITT) analysis set of 568 patients, corresponding to an SMD (Hedges’ g) of 0.300 [5,11,13]. The primary endpoint was statistically significant (p=0.005), qualifying for the 0.001≤p<0.01 adjustment tier. After applying the 1.10 multiplier, the adjusted efficacy score was 0.330.

Engagement

The protocol-specified adherence threshold was task completion on at least 70% of days. Engagement was high, with patients completing a mean of 89.7% of scheduled daily tasks during the 12-week intervention [11,13]. The engagement rate was normalized to a 0-1 scale, yielding an engagement score of 0.897.

Evidence Quality

The ReMMi-D trial was a randomized, double-blind, sham-controlled study meeting all criteria for Class I evidence, including prospective enrollment, concealed allocation, objective outcome assessment, and ≥80% patient retention [11,13]. Accordingly, the evidence quality score assigned was 1.00.

Safety

No treatment-emergent adverse events were attributed related to CT-132 during the pivotal trial [11,13]. Therefore, a safety penalty of 0.00 was applied.

cDTI

The composite index for CT-132 was calculated as: 

$\text{cDTI} = 0.330 \times 0.897 \times 1.00 \times (1 - 0.00) = 0.296$. 

Thus, CT-132 achieved a cDTI score of 0.296.

Comparative summary

CT-132 achieved a cDTI score of 0.296, 12.87 times higher than reSET-O’s score of 0.023. This substantial difference reflects superior clinical efficacy, higher user engagement, greater methodological rigor, and a more favorable and known safety profile for CT-132.

**Figure 1:**
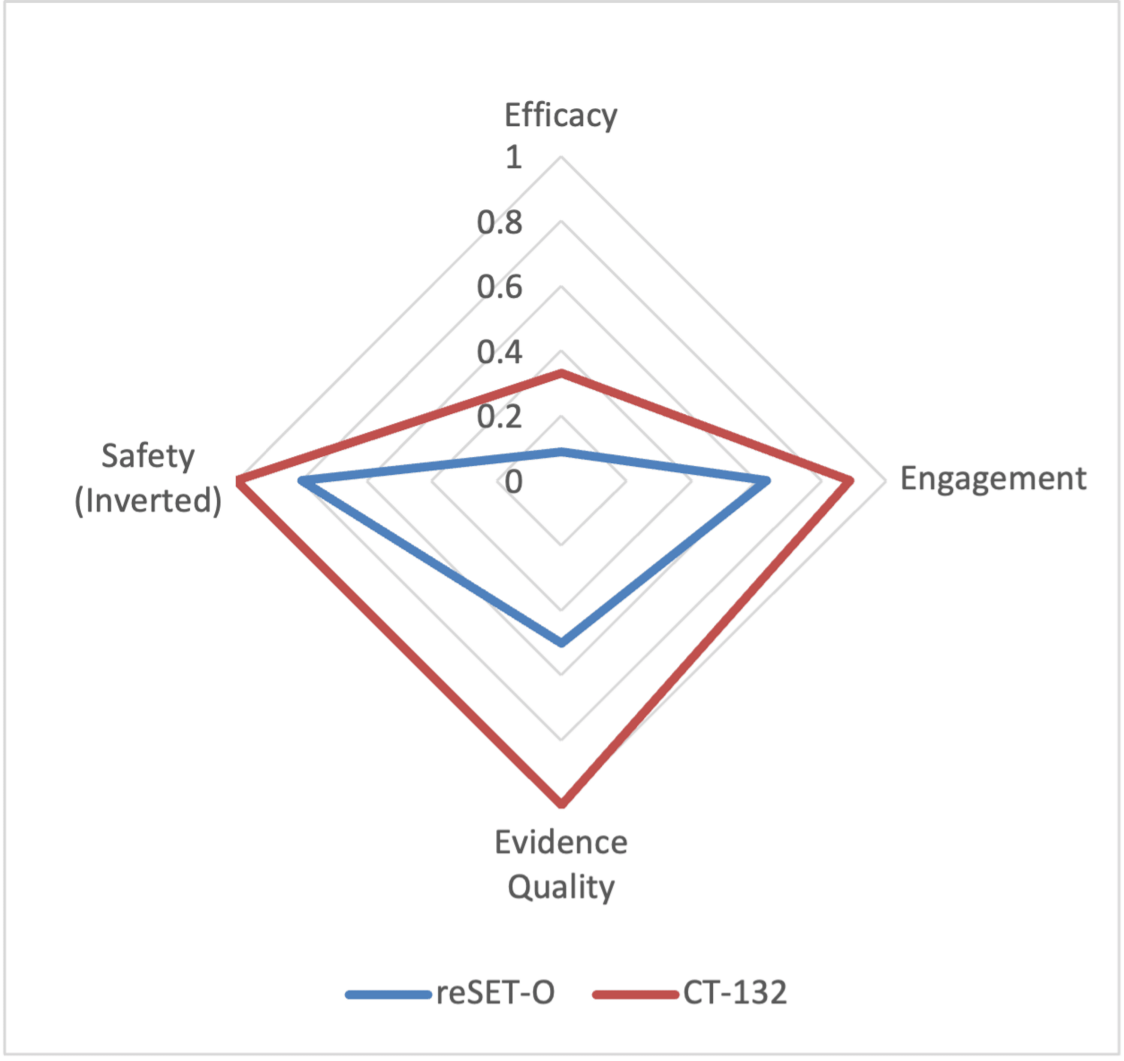
Radar Plot of cDTI Domain Scores for reSET-O and CT-132 Radar plot (spider diagram) comparing four domain scores used to calculate the Composite Digital Therapeutic Index (cDTI) for reSET-O and CT-132. Domains include efficacy (standardized mean difference adjusted by statistical significance), engagement (proportion achieving a level of therapeutic completion), evidence quality (based on adapted American Academy of Neurology Class of Evidence criteria), and inverted safety penalty (1 minus safety penalty based on treatment-related/emergent adverse events), where 1 indicates no device-related adverse events and lower scores reflect greater risk. CT-132 demonstrated superior scores across all domains compared to reSET-O.

Radar plots illustrating the domain-specific performance profiles of reSET-O and CT-132 are presented in Figure 1. Tabulated domain scores and final cDTI values are presented in Table 6.

**Table 6:** Domain Scores and cDTI for reSET-O and CT-132 Comparison of individual domain scores of adjusted efficacy (standardized mean difference, SMD, corrected for statistical significance), engagement (proportion normalized to 0–1), evidence quality (based on adapted American Academy of Neurology Class of Evidence), and safety penalty, along with final calculated Composite Digital Therapeutic Index (cDTI) values for prescription digital therapeutics (PDTs) reSET-O and CT-132. CT-132 demonstrated higher scores across all domains, resulting in a cDTI 12.87 times greater than reSET-O.

PDT	Adjusted Efficacy	Engagement	Evidence Quality	Safety Penalty	cDTI
reSET-O	0.0895	0.630	0.50	0.20	0.023
CT-132	0.330	0.897	1.00	0.00	0.296

## Discussion

Our analysis demonstrates that the cDTI framework can meaningfully differentiate PDTs based on clinical trial performance, patient engagement, trial rigor, and safety profile. While both reSET-O and CT-132 have demonstrated meeting regulatory standards for FDA market authorization, the magnitude and multidimensional quality of their outcomes vary considerably.

reSET-O exhibited limitations including non-statistically significant primary outcome findings, modest sample size, and an open-label study design without patient blinding and with a usual-care control, and lack of systematic treatment-related and -emergent adverse event reporting, culminating in a cDTI score of just 0.023. It should be noted that the FDA indication for use for reSET-O is to "increase retention of patients with opioid use disorder (OUD) in outpatient treatment" which was not the primary endpoint of the registration trial. Further, the reSET-O app was later developed from this academic web-based intervention in the study. By contrast, CT-132 benefits from a highly statistically and clinically significant clinical outcomes, larger sample size, robust double-blinded randomized controlled trial (RCT) design with sham, high therapeutic adherence with predefined criteria, and low incidence of systematically collected and reported safety events (zero PDT-related treatment emergent adverse events), all of which contribute to its relatively higher cDTI score of 0.296. The FDA indication for use for CT-132 is akin to the target of pharmacological agents, "for the preventive treatment of episodic migraine", and was developed smartphone app first. This illustrates how the cDTI framework captures differences across multiple therapeutic dimensions not apparent through effect sizes alone.

Importantly, this discrepancy aligns with stakeholder assessments. The ICER 2020 review of reSET-O found the evidence insufficient to determine long-term effectiveness, citing limitations in study blinding, follow-up, and generalizability [16,17]. Meanwhile, CT-132 data from the ReMMi-D trial offers a more contemporary model of digital therapeutic development, featuring a sham-controlled comparator, high patient adherence, and rigorous outcome measurement. These elements align well with regulatory expectations for software as a medical device (SaMD) products as evidenced by CT-132 earning FDA Breakthrough Device Designation during development [18].

Unlike traditional HTA tools or mobile health app ratings, the cDTI enables structured cross-PDT comparisons using quantitative inputs, consistent weighting, and an easy-to-interpret single value between 0 and 1. It addresses gaps in frameworks like Mobile App Rating Scale [24], Digital Medicine Society's V3 Framework [25], and the Digital Therapeutics Alliance DTx Evaluation Toolkit [26], which lack comparative scoring capacity. cDTI uniquely includes both clinical evidence and patient engagement, a multidomain feature increasingly demanded by payers and regulators per ICER and the Peterson Health Technology Institute [27]. Furthermore, the cDTI allows stakeholders to benchmark products in a reproducible, domain-transparent manner. 

The cDTI framework provides clinicians, payers, and regulatory bodies with a transparent and reproducible metric to evaluate PDTs. As the digital therapeutic landscape expands, health systems need objective tools to inform coverage decisions, formulary inclusion, and guideline integration. For instance, two PDTs cleared for the same indication may differ substantially in trial quality, adherence, or safety profile. The cDTI enables differentiation not based on marketing claims, but on standardized, evidence-based metrics.

Improvements to framework

To ensure the cDTI remains a scalable, clinically meaningful, and future-proof tool, we propose several refinements organized by category in Table 7.

**Table 7:** Proposed Enhancements to the cDTI Framework This table summarizes future refinements to the Composite Digital Therapeutic Index (cDTI) framework, organized by domain, to ensure scalability, clinical relevance, and alignment with evolving digital therapeutic standards. PDT: Prescription digital therapeutics, AAN CoE: American Academy of Neurology Class of Evidence, TEAE: treatment-emergent adverse event, HTA: health technology assessments

Category	Proposed Enhancement	Original Description
Efficacy Enhancements	Dose-Response / Engagement Gradient Adjustment	Several digital therapeutics demonstrate a correlation between engagement level and clinical benefit, such as greater symptom improvement with higher module completion rates. Incorporating a dose-response or engagement gradient adjustment could reward interventions with demonstrable engagement-response curves. For example, PDTs showing a statistically significant improvement gradient based on usage quartiles or time-on-task could receive an efficacy multiplier or increased engagement weighting, helping distinguish meaningful engagement from superficial app use.
	Durability Adjustment	Future versions of the cDTI may incorporate a modifier for sustained therapeutic effect. PDTs demonstrating benefits persisting beyond 12 weeks (or longer than the pivotal trial treatment duration) could receive a score multiplier (e.g., +10%) to reward durability and long-term clinical value.
Engagement Enhancements	Enhancing and Refining Engagement Metrics	Recognizing that engagement is a multidimensional construct encompassing frequency, duration, intensity, and depth of interaction, the current cDTI uses completion-based adherence as a pragmatic proxy. However, future iterations may incorporate richer digital biomarkers, such as attention patterns and depth of task interaction, to better capture cognitive involvement, emotional resonance, and user engagement quality.
	Dynamic Adherence Weighting / Adjustment	Engagement was defined as the mean proportion of scheduled therapeutic tasks completed during the intervention period, based on objective adherence metrics reported in pivotal trials. Future versions of the framework may incorporate dynamic weighting or contextual adjustments by therapeutic domain, intervention complexity, or disease area to enhance clinical relevance.
Evidence Quality Enhancements	PDT-Specific Evidence Quality Framework	While the adapted AAN CoE framework offers a structured and reproducible method for trial quality assessment, future iterations may develop a bespoke PDT-specific grading framework. This would better address unique digital therapeutic features such as adaptive content, engagement variability, and real-world scalability.
	Integration of Real-World Evidence	Future adaptations could incorporate post-market safety surveillance, patient-reported outcomes, and real-world effectiveness data to supplement traditional trial-based evidence quality scoring.
Safety Enhancements	Severity-Weighted Safety Scoring	The current cDTI treats TEAEs uniformly. Future versions could apply differentiated penalties based on severity, allowing greater penalty for moderate or severe events and smaller penalties for mild TEAEs.
	Post-Market Surveillance Integration	Incorporating longitudinal safety data from real-world use and post-authorization studies would further strengthen safety assessment beyond the pivotal trial setting.
Composite Score and Structural Enhancements	Domain Weighting	While the current cDTI framework equally weights efficacy, engagement, evidence quality, and safety, future iterations could apply differential weights reflecting clinical, payer, and patient priorities. For instance, efficacy could carry a weight of 0.4, evidence quality 0.3, engagement 0.2, and safety 0.1 to ensure that minimal efficacy or poor evidence quality cannot be offset by high adherence alone.
	Score Tiers for Interpretation	To facilitate interpretability, cDTI scores could be grouped into qualitative tiers (e.g. greater than 0.20 as strong therapeutic value; between 0.10 and 0.20 as moderate, conditionally valuable; and less than 0.10 as low or uncertain value).​​
	Handling of Missing Data	While current cDTI scoring uses conservative imputation (default values) to penalize missing data, future versions may refine this approach using uncertainty bands, sensitivity scores, or domain-specific penalization to better represent data confidence.
	Expanded Visualization Tools	Radar plots could be complemented with bar charts, composite indices, or uncertainty heatmaps to visually depict strengths, weaknesses, and variability across PDTs.
	Alignment with External Frameworks	Mapping the cDTI to existing evaluation frameworks (e.g., ICER assessments, FDA Breakthrough Device criteria, NICE Digital Health guidelines) would ensure alignment with global payer and regulatory priorities.
	Scalability Across Regulatory Classes	Although currently designed for 510(k) and De Novo clearances, the cDTI can be expanded to include PDTs supported by real-world evidence, observational studies, or combination drug-digital therapies ("smart medicines").
Future Domain Additions	Clinical Utility Index	We propose developing a Clinical Utility Index (CUI) to complement the cDTI. While the cDTI captures trial-based performance, the CUI would integrate patient-reported outcomes (PROs) such as the Patient Global Impression of Change (PGI-C) to reflect patient-perceived benefit. For example, the cDTI could be multiplied by a normalized PRO score to prioritize interventions perceived as meaningfully helpful by patients.
	Equity, Digital Inclusion, and Cost-Effectiveness	As digital health expands, future versions (e.g., cDTI 2.0) may integrate domains reflecting health equity, digital literacy, inclusion barriers, and cost-effectiveness to improve HTA relevance to public health decision-making.

Limitations

This study presents a conceptual framework applied to only two PDTs and is intended as a proof-of-concept rather than a definitive comparative analysis. Conservative imputation of missing data, particularly engagement and safety, may misrepresent true performance. The cDTI relies on published and regulatory sources, which may be subject to reporting bias or incomplete information. SMD as a measure of efficacy assumes a normal distribution of treatment effects and may not capture clinically significant thresholds across diverse outcome measures. Future iterations should address these limitations by incorporating real-world data and broader stakeholder input.

The use of conservative imputation for missing engagement and safety data may influence cDTI scores, particularly if applied to multiple products. While imputation was used sparingly and according to a predefined hierarchy, its impact on score variability warrants further investigation. Future versions of the framework should incorporate sensitivity analyses and uncertainty bands to better represent confidence in domains with incomplete data.

We are planning future studies that would extend this framework to all FDA-cleared PDTs to establish field-wide benchmarks and support rational, evidence-based adoption. Further, we will assess its responsiveness to emerging evidence and explore adaptations for drug+PDT smart medicines and international digital therapeutic products. Integration of additional domains as outlined above such as equity impact, cost-effectiveness, and durability of clinical effect may further enhance the framework’s utility for therapeutic assessment.

## Conclusions

The cDTI provides a structured and reproducible framework to evaluate PDTs across multiple domains. In this proof-of-concept application, CT-132 demonstrated a higher overall therapeutic index than reSET-O due to superior outcomes, engagement, trial design, and safety. The cDTI may serve as a decision-support tool for health systems, HTAs, regulatory agencies, and payors, particularly as more PDTs seek reimbursement and clinical integration into systems of healthcare. Future validation of the cDTI against real-world outcomes, healthcare utilization, and longitudinal adherence and engagement metrics is planned to assess external validity.
